# Dens Invaginatus as a Biomechanical Risk Modifier in Dental Trauma: Finite Element Analysis

**DOI:** 10.1111/edt.70078

**Published:** 2026-05-04

**Authors:** Emine Şuranur Ayaz, Sema Aydınoğlu, Dilara Nil Günaçar, Ayberk Dizdar, Ecren Uzun Yaylaci, Murat Yaylaci, Mukaddes Yerebakan

**Affiliations:** ^1^ Recep Tayyip Erdoğan University, Faculty of Dentistry, Department of Pediatric Dentistry Rize Turkey; ^2^ Recep Tayyip Erdoğan University, Faculty of Dentistry, Department of Oral and Maxillofacial Radiology Rize Turkey; ^3^ Department of Biomedical Engineering, Kocaeli University Kocaeli Turkey; ^4^ Faculty of Fisheries, Recep Tayyip Erdogan University Rize Turkey; ^5^ Department of Civil Engineering Recep Tayyip Erdogan University Rize Turkey; ^6^ Turgut Kıran Maritime Faculty, Recep Tayyip Erdogan University Rize Turkey; ^7^ Dijitalpark Teknokent, Murat Yaylacı‐Luzeri R&D Engineering Company Rize Turkey; ^8^ Rize Oral and Dental Health Center Rize Turkey

**Keywords:** Dentoalveolar trauma, finite element analysis, stress, tooth abnormalities

## Abstract

**Introduction/Aims:**

Traumatic dental injuries are common in childhood and may adversely affect functional and psychosocial well‐being. Dens invaginatus, a developmental anomaly caused by the infolding of the enamel organ during odontogenesis, alters tooth morphology and may influence biomechanical behavior under traumatic loading. This study aimed to evaluate stress distribution and deformation patterns in permanent maxillary lateral incisors with different types of dens invaginatus using three‐dimensional dynamic finite element analysis and to compare them with a morphologically normal tooth.

**Material and Methods:**

Finite element models representing four dens invaginatus configurations of maxillary lateral incisors (#22) were analyzed under dynamic impact loading generated by a rigid steel sphere (radius 10 mm; velocity 10 m/s). Horizontal and vertical impact scenarios were simulated. In addition, a static occlusal force of 100 N was applied to the palatal surface to reproduce functional contact. Stress distribution and deformation patterns were compared with those of a morphologically normal incisor model.

**Results:**

Dens invaginatus models showed substantially higher biomechanical responses than the normal tooth under both loading directions. Tooth stresses increased by approximately 110–130%, deformation values nearly doubled, and microstrain levels rose progressively with anomaly severity. The lowest responses were observed in the Type I model, whereas the Type IIIb configuration consistently demonstrated the highest stress, deformation, and strain values. Principal stresses in surrounding structures were also elevated, increasing by roughly 40–70%.

**Conclusions:**

Dens invaginatus markedly modifies the biomechanical response of lateral incisors subjected to traumatic forces. Both internal morphology and impact direction influence stress distribution, with vertical loading generating greater stresses. Increased biomechanical susceptibility was evident even in the mildest form (Type I), while the most severe configuration (Type IIIb) exhibited the highest stress and deformation responses, indicating that dens invaginatus should be regarded as a clinically relevant biomechanical risk factor in dental trauma assessment.

## 1 Introduction

Traumatic dental injuries are a common oral health condition in childhood and represent a significant global public health problem, affecting approximately 15%–23% of individuals in both primary and permanent dentitions while negatively influencing children's quality of life [1]. Appropriate and timely intervention is critical for the prognosis of traumatized teeth. Delays in treatment or inappropriate therapeutic approaches may adversely affect prognosis and lead to tooth loss, resulting in substantial functional, esthetic, economic, psychological and social consequences for affected children [2].

Dental anomalies are defined as disorders characterized by deviations from the normal developmental course in the shape, structure, number, size and position of teeth [3]. Abnormalities observed after the completion of tooth development are referred to as acquired dental anomalies, whereas those occurring during ongoing tooth development are defined as developmental dental anomalies [4]. Dens invaginatus, which is classified among developmental dental anomalies within the group of morphological disturbances, occurs as a result of the invagination of the enamel organ into the dental papilla during odontogenesis [5]. Previous studies have reported that the incidence of dens invaginatus ranges from 0.25% to 26.5% among examined individuals [6]. Radiographic examinations may reveal an invagination confined to the crown or extending into the root region, appearing as a radiolucent area surrounded by radiopaque enamel. When this invagination communicates with the oral environment, it may facilitate the retention of microorganisms and food debris, thereby increasing the risk of infection [7]. Dens invaginatus is most commonly observed in permanent teeth, particularly in maxillary lateral incisors [8]. It may also occur, in decreasing order of frequency, in maxillary central incisors, canines, premolars and more rarely, molars. Although uncommon, cases occurring in supernumerary teeth have also been reported in the literature [9].

Teeth and the surrounding tissues are subjected to different loading conditions under traumatic forces; however, the biomechanical behavior of traumatic events and their effects on adjacent tissues cannot be precisely determined due to case‐dependent variables [10]. Numerous factors influence the severity of traumatic dental injuries and contribute to the biomechanical complexity of trauma, including the magnitude and direction of the applied force, the site of force application, the elasticity and geometry of the impacting object and the resistance exhibited by the teeth and surrounding tissues [11]. To accurately elucidate the biomechanical response of dentoalveolar structures to trauma and to guide clinical decision‐making, a detailed evaluation of stress distribution induced by traumatic forces within the teeth and adjacent tissues is required [12].

Direct experimental investigation of the biomechanical behavior of tissues subjected to traumatic forces is generally limited by ethical concerns. Accordingly, conducting stress analyses using models that represent the biomechanical properties of living tissues is considered a more realistic and applicable approach [13]. In this context, finite element analysis (FEA) enables the performance of such investigations. This method allows the creation of a digitally reconstructed, anatomically realistic model of the structure of interest or a specific region of living tissues within a computer environment. Consequently, physical events such as trauma can be simulated digitally, making it possible to predict the structural and mechanical behavior of the related systems [14].

To the best of our knowledge, although dens invaginatus significantly alters tooth morphology and may influence its biomechanical response, no previous studies have examined how different configurations of this anomaly affect stress distribution and deformation patterns during traumatic loading. The aim of this study was to evaluate the biomechanical behavior of maxillary lateral incisors with four different dens invaginatus configurations using FEA and to compare their stress distribution and deformation patterns with those of anatomically normal lateral incisors. The null hypothesis (H_0_) of this study was that there would be no differences in stresses and deformations generated during traumatic loading among the four dens invaginatus lateral incisor models and the lateral incisor model with normal morphology. In addition to addressing a gap in the literature, the present study introduces a novel perspective by conceptualizing dens invaginatus not only as a morphological anomaly but also as a potential biomechanical risk modifier in traumatic dental injuries.

## 2 Materials and Methods

### 2.1 Geometry

A publicly available dataset containing anatomical images and computed tomography (CT) sections from the Visible Human Project (The National Library of Medicine, FACT SHEETS, Office of Communications and Public Liaison National Library of Medicine, Maryland, USA; https://data.lhncbc.nlm.nih.gov/public/Visible‐Human/Female‐Images/index.html) was used to construct a three‐dimensional finite element model. The dataset provides high‐resolution anatomical information and has been widely used in biomechanical modeling studies. The data were processed using 3D Slicer software (Brigham and Women's Hospital, Boston, MA, USA; https://www.slicer.org/) following the method described by Fedorov et al. [15]. Segmentation was performed in 3D Slicer using Hounsfield unit (HU) thresholding to generate three‐dimensional models, which were subsequently exported for further processing and FEA. Segmentation of the CT data was primarily used to reconstruct the maxillary bone, whereas dental morphology was subsequently generated using anatomical references [16]. An HU range of 426.50–3193.04 was selected according to the density characteristics of the target tissues, enabling effective differentiation of the anatomical structures of interest while reducing the inclusion of surrounding non‐relevant regions. The HU range was determined through an iterative optimization process in which multiple threshold intervals were evaluated. The resulting three‐dimensional reconstructions were assessed for anatomical consistency and the final threshold values were selected to preserve the structural integrity and morphological detail of the target tissues while minimizing the inclusion of surrounding non‐relevant regions.

The three‐dimensional model was imported into SpaceClaim software (Version 22.0; ANSYS Inc., Canonsburg, PA, USA) for geometric reconstruction. The maxillary bone and dental structures were modeled and a cortical bone layer with a thickness of 2 mm was generated by applying a uniform offset to the maxillary bone model [17]. The internal surface of the resulting cortical bone was subsequently used as a reference boundary to define the trabecular bone region. Anatomical tooth morphology was reconstructed according to Wheeler's Dental Atlas [16]. Because dens invaginatus cases with identical crown and root dimensions could not be obtained for each type, both the morphologically normal permanent lateral incisor and the four dens invaginatus configurations were digitally reconstructed. This strategy was adopted to ensure geometric standardization and to allow reliable comparison among the models. Periodontal ligament layers with a thickness of 0.25 mm were generated along the external surfaces of the teeth [17].

Because the traumatic load was applied to tooth #22, all analyses were performed using a left maxillary hemimaxilla model. This segment included seven teeth together with their periodontal ligament and supporting alveolar bone. Four separate models representing different dens invaginatus configurations were constructed for tooth #22. This modeling strategy was adopted to reduce computational cost while ensuring consistency across simulations. Bonded contact conditions were assigned to all interfaces within the biomodels.

According to Oehlers' classification of dens invaginatus, [18] four anatomical configurations were reconstructed for finite element modeling. In Type I, the invagination is confined to the crown and does not extend beyond the cemento‐enamel junction. In Type II, the invagination extends apically beyond the cemento‐enamel junction into the root canal system but remains within the root and does not create a separate communication with the periodontal tissues. Type III represents the most severe form, in which the invagination penetrates through the root. In Type IIIa, the invagination extends through the root and communicates laterally with the periodontal ligament space without direct pulpal involvement. In contrast, Type IIIb is characterized by an invagination that traverses the root and opens at the apical foramen, forming a direct communication between the oral environment and the periodontal tissues [18]. These anatomical configurations were digitally reconstructed using SpaceClaim software to generate the corresponding finite element models (Figure 1).

**FIGURE 1:**
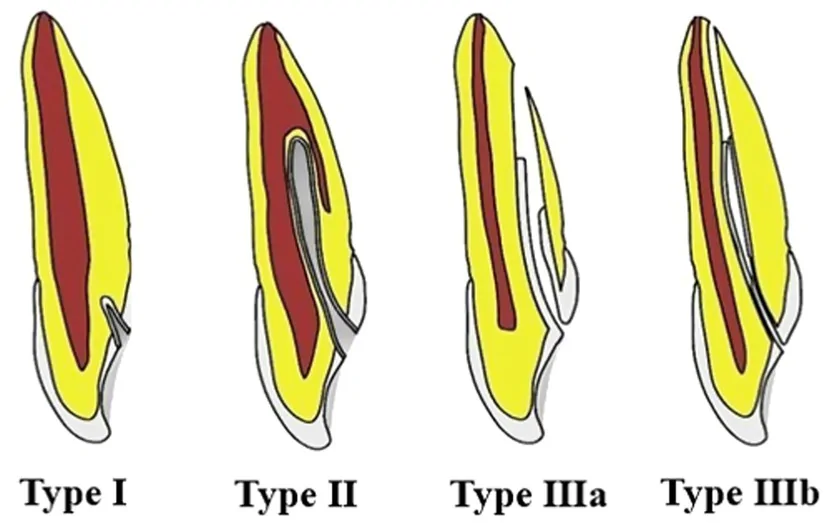
Schematic representation of four dens invaginatus configurations reconstructed according to Oehlers' classification (Types I, II, IIIa, and IIIb) for finite element modeling.

### 2.2 Mesh Structure

The solid models were prepared for FEA and mesh optimization and numerical simulations were performed using ANSYS Workbench software (ANSYS Inc., Canonsburg, PA, USA, 2022). A mesh convergence analysis was conducted by applying different element sizes to the model. The analysis was initiated with an element size of 1.0 mm, which was gradually reduced in 0.1 mm increments to 0.5 mm. Based on the convergence results, mesh independence was achieved at an element size of 0.5 mm (Figure 2).

**FIGURE 2:**
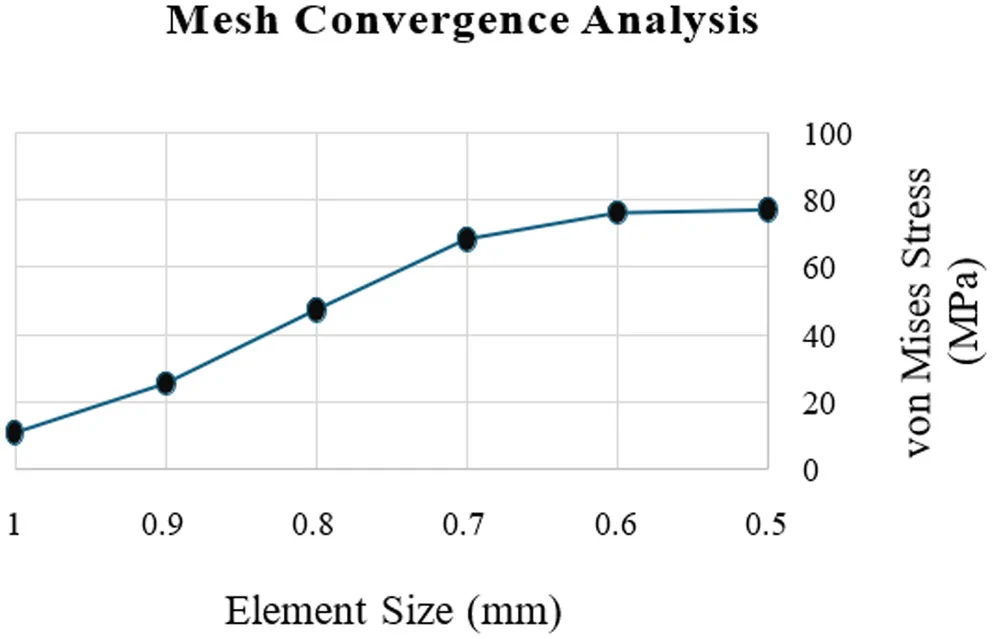
Mesh convergence analysis showing the change in maximum von Mises stress with decreasing element size.

Following the convergence study, the models were discretized using quadratic tetrahedral elements (SOLID92), which are suitable for representing complex anatomical geometries. To optimize numerical accuracy and computational efficiency, different mesh densities were applied to individual model components. Element sizes were defined as 0.075 mm for the teeth, 1 mm for the maxillary structure, and 4 mm for the steel sphere. The elements were defined with full three‐dimensional degrees of freedom, allowing translation and rotation along the x, y, and z axes. The final mesh configuration consisted of approximately 542.304 nodes and 356.779 elements (Figure 3).

**FIGURE 3:**
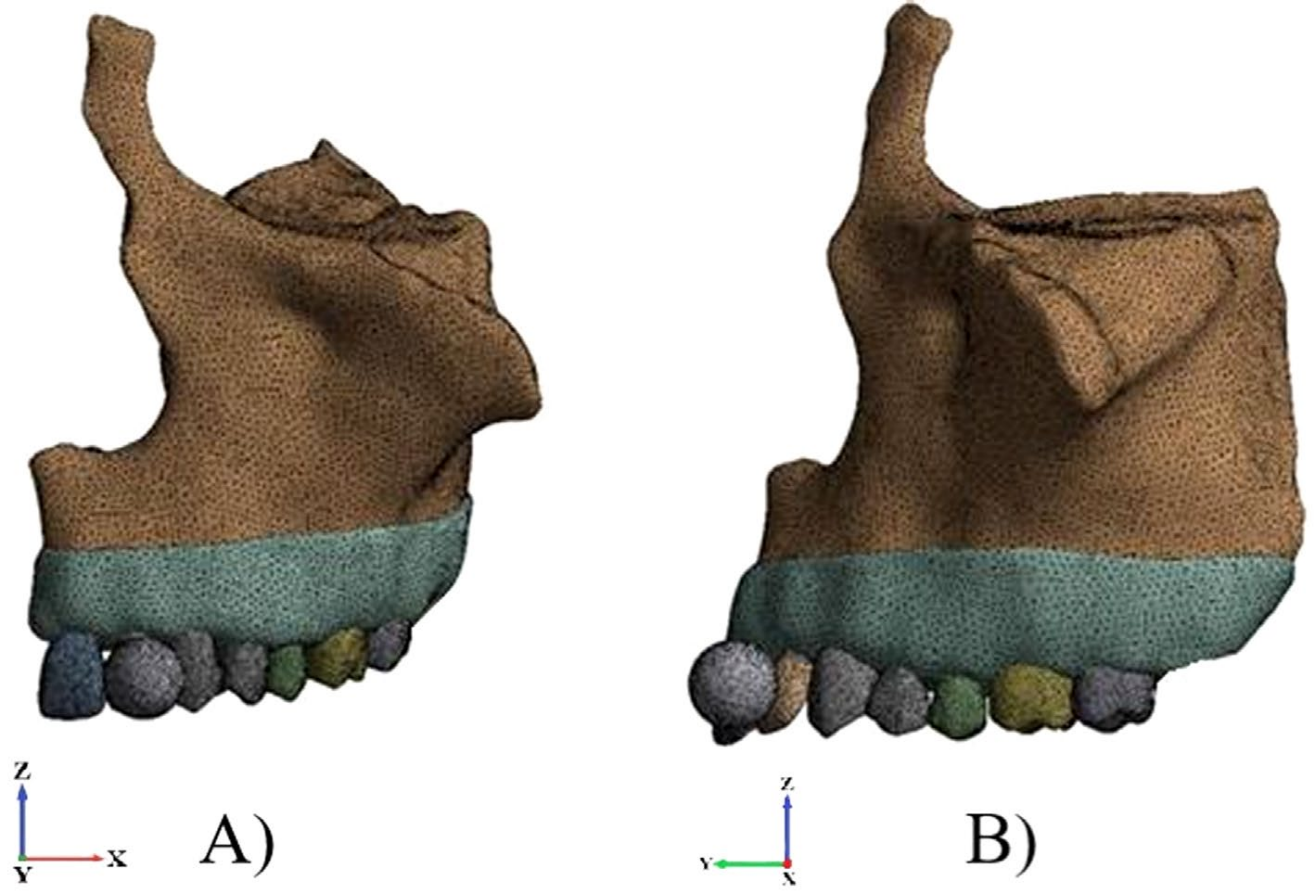
Finite element mesh of the maxillary model illustrating the applied loading conditions: **(A)** horizontal and **(B)** vertical directions.

### 2.3 Material Properties

To accurately evaluate the biomechanical response of dental and supporting tissues under the applied loads, the mechanical properties of each structure were defined prior to analysis. Material parameters, including Young's modulus, Poisson's ratio, and density, were assigned according to values reported in the literature and implemented in the finite element model [19, 20, 21, 22, 23, 24] (Table 1). All structures were assumed to be homogeneous, isotropic, and linearly elastic, consistent with commonly adopted assumptions in dental finite element studies.

**TABLE 1:** Mechanical properties of the materials incorporated into the finite element model.

Structure	Elastic Modulus (MPa)	Density (g/cm3)	Poisson's ratio
Enamel^19^	84.100	2.14	0.30
Dentin^20^	18.600	2.97	0.30
Pulp^21^	6.8	1	0.45
Periodontal Ligament^22^	50	0.95	0.45
Trabecular Bone^23^	1.400	0.70	0.31
Cortical Bone^23^	13.700	2.00	0.33
Steel^24^	200.000	0.95	0.30

### 2.4 Boundary and Loading Conditions

To simulate traumatic effects, dynamic impact loading was applied by modeling the collision of a rigid steel sphere with a radius of 10 mm against the teeth at a velocity of 10 m/s [25, 26] (Figure 4). Two loading scenarios were considered:

**FIGURE 4:**
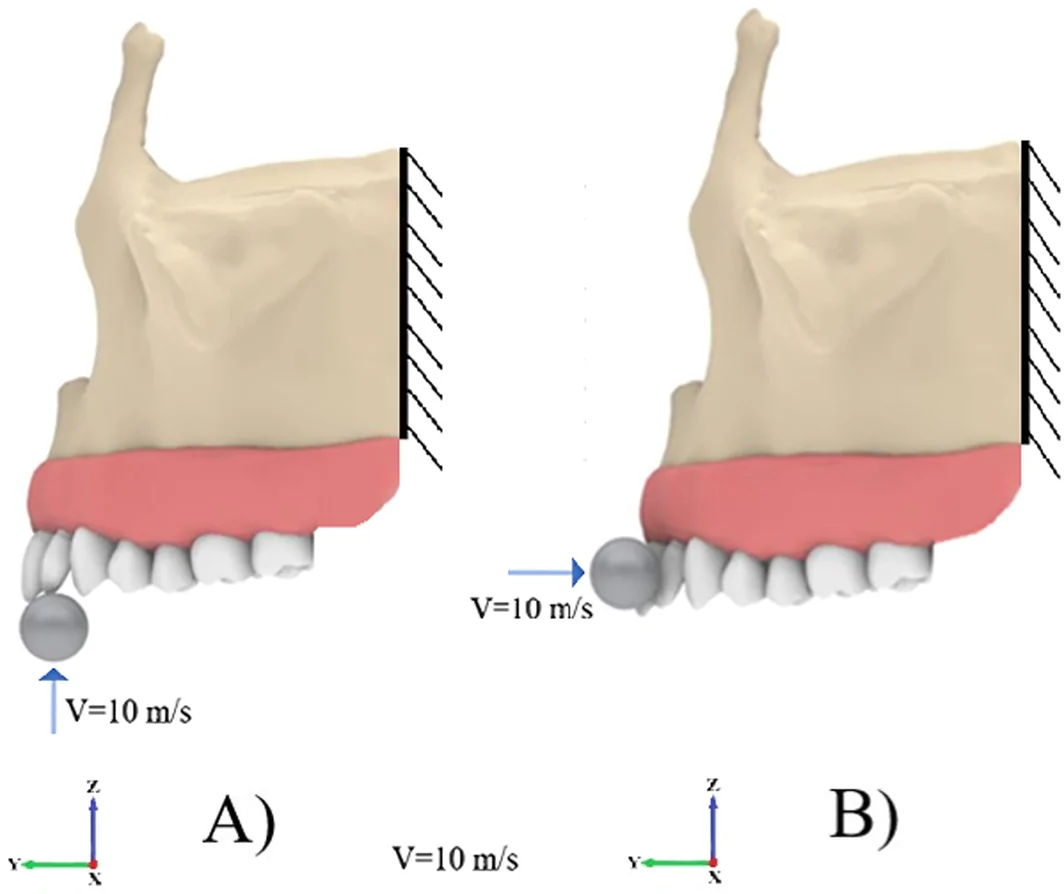
Boundary and loading conditions applied in the finite element simulations: **(A)** *Scenario V*: Vertical impact directed toward the incisal region and **(B)** *Scenario H*: Horizontal impact directed toward the buccal surface, both simulated using a rigid sphere with a velocity of 10 m/s.

*Scenario H*: The impact load was applied horizontally to the midpoint of the buccal surface of the maxillary lateral incisor based on anatomical reference points.

*Scenario V*: The impact load was applied vertically to the midpoint of the incisal edge of the maxillary lateral incisor based on anatomical reference points.

In addition, to evaluate the influence of occlusal contact, a static bite force of 100 N was simultaneously applied to the palatal surface of the lateral incisors [25].

All simulations were performed in ANSYS Workbench under the specified boundary and loading conditions and the resulting stress and deformation fields were analyzed.

## 3 Results

In this study, a series of finite element simulations were performed to evaluate the biomechanical response of maxillary lateral incisors with different dens invaginatus configurations under traumatic loading conditions. The analysis aimed to assess stress distribution, deformation patterns, displacement, and microstrain values in four dens invaginatus models and to compare them with those of a lateral incisor exhibiting normal morphology. The findings revealed distinct mechanical responses among the models, indicating significant differences in stress concentrations and deformation behavior depending on the dens invaginatus configuration and loading direction.

Cross‐sectional views of the stress distributions in the normally structured lateral incisor and the dens invaginatus tooth models at the peak of horizontal and vertical impact loading are shown in Figure 5. Stress values were evaluated according to the von Mises criterion (MPa) and illustrated using a linear color scale, where blue represents lower values and red represents higher values. In all models, the highest stress concentrations were primarily localized at the contact interface with the steel ball, with secondary stress accumulation observed in the cervical region. Stress values gradually decreased with increasing distance from the impact site, and vertical impact loading produced higher stress values than horizontal loading. Maximum von Mises stress values increased markedly with the severity of dens invaginatus, rising from approximately 76–78 MPa in the normally structured tooth to about 163 MPa under horizontal loading and 179 MPa under vertical loading in the Type IIIb model, corresponding to increases of roughly 110% and 130%, respectively. Overall, the dens invaginatus models exhibited greater stress concentrations than the tooth with normal morphology, with the Type IIIb configuration demonstrating the highest stress values among all models.

**FIGURE 5:**
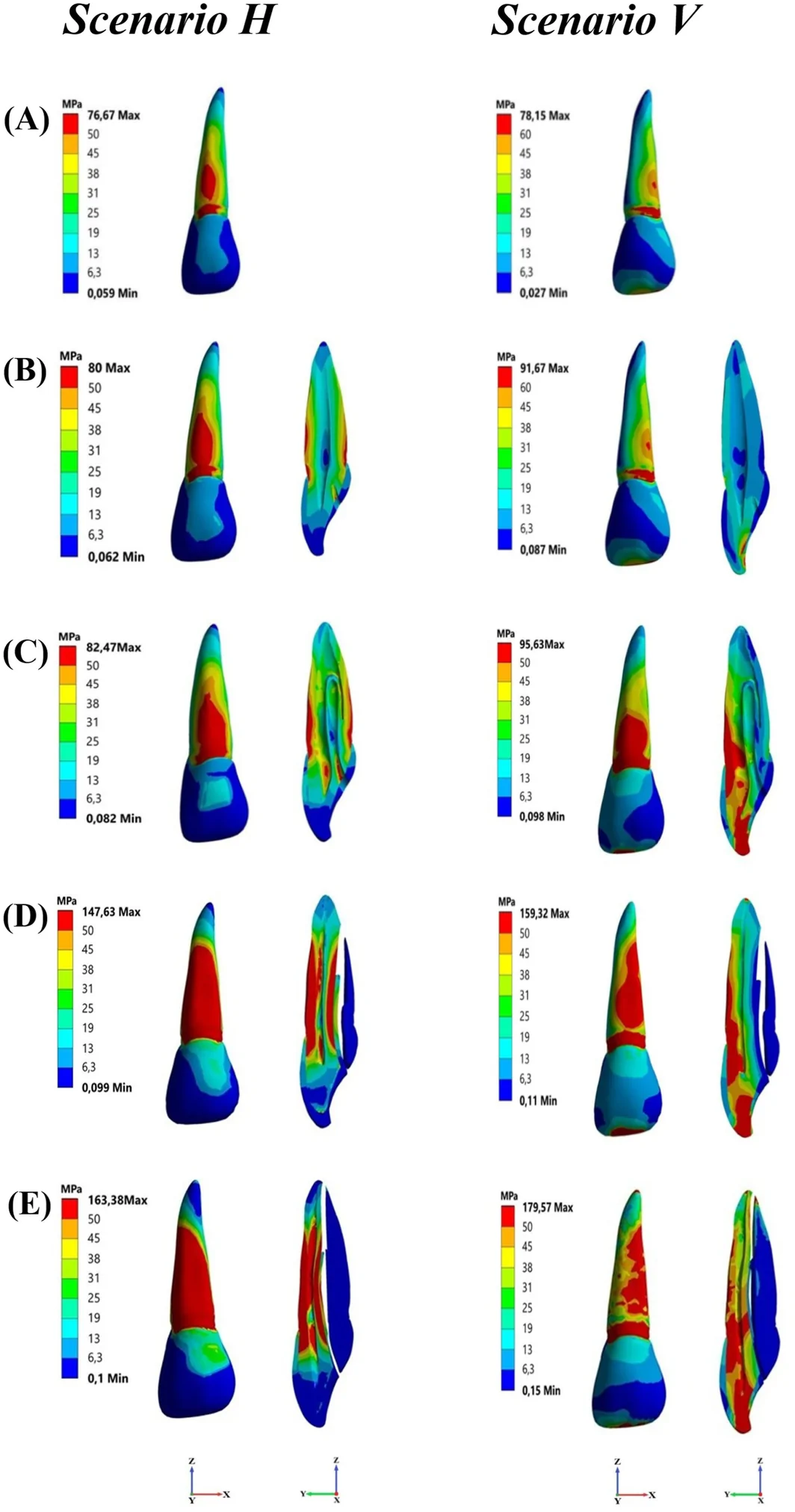
Von Mises stress distributions in tooth models under horizontal (*Scenario H*) and vertical (*Scenario V*) impact loading directed toward the buccal surface: **(A)** Tooth with normal morphology, **(B)** Type I dens invaginatus, **(C)** Type II dens invaginatus, **(D)** Type IIIa dens invaginatus, **(E)** Type IIIb dens invaginatus.

The principal stress distributions in the alveolar bone under both loading conditions are presented in Figure 6. Under horizontal impact loading, stress concentrations were primarily localized in the alveolar bone surrounding the cervical and apical regions of the tooth, whereas vertical impact loading produced a more pronounced stress accumulation in the cervical region. Both maximum and minimum principal stress values recorded in the dens invaginatus models were higher than those observed in the tooth with normal morphology. Among the dens invaginatus models, the highest stress values were identified in the Type IIIb configuration. Compared with the normally structured tooth, the Type IIIb model exhibited higher principal stress values under both loading directions, increasing from approximately 37 MPa to about 62 MPa under horizontal loading and from approximately 52 MPa to about 72 MPa under vertical loading, corresponding to increases of roughly 68% and 39%, respectively.

**FIGURE 6:**
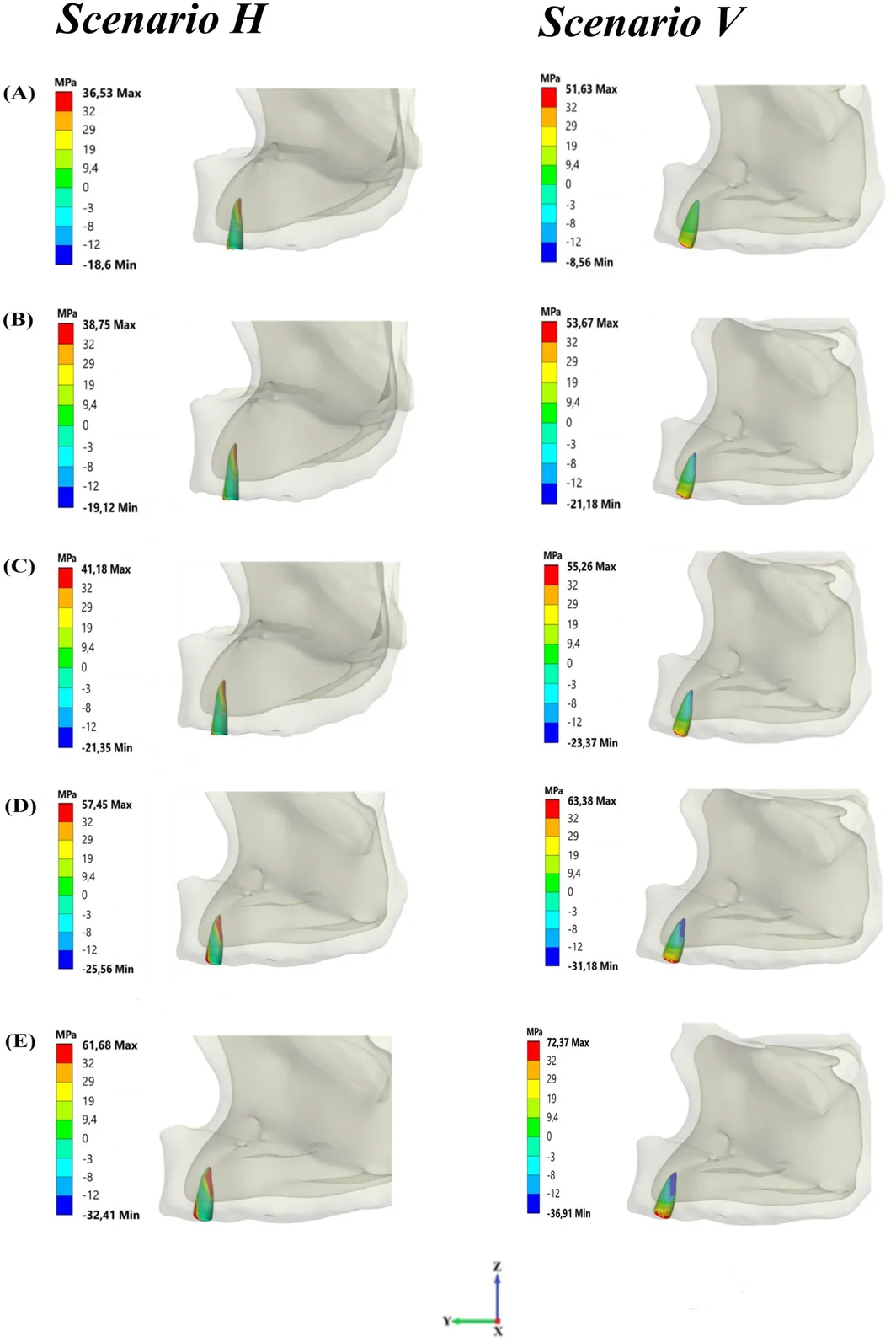
Principal stress distributions in the alveolar bone under horizontal *(Scenario H)* and vertical *(Scenario V)* impact loading directed toward the buccal surface: **(A)** Tooth with normal morphology **(B)** Type I dens invaginatus **(C)** Type II dens invaginatus **(D)** Type IIIa dens invaginatus **(E)** Type IIIb dens invaginatus.

The total deformation patterns under both loading conditions are presented in Figure 7. Horizontal and vertical impact loading produced distinct mechanical responses in the tooth models. Maximum deformation was predominantly concentrated at the impact contact area and gradually decreased with increasing distance from this region. In the normally structured tooth as well as in the Type I and Type II dens invaginatus models, deformation was primarily localized at the incisal edge of the crown. In contrast, the Type IIIa and Type IIIb models exhibited a more extensive distribution of deformation across the crown. Overall, the dens invaginatus models demonstrated higher deformation values than the normally structured tooth, and deformation magnitudes were greater under vertical impact loading. Maximum deformation increased progressively with the severity of dens invaginatus, rising from approximately 0.19 mm (horizontal) and 0.25 mm (vertical) in the normally structured tooth to about 0.42 mm and 0.56 mm, respectively, in the Type IIIb model, representing nearly a two‐fold increase in deformation magnitude.

**FIGURE 7:**
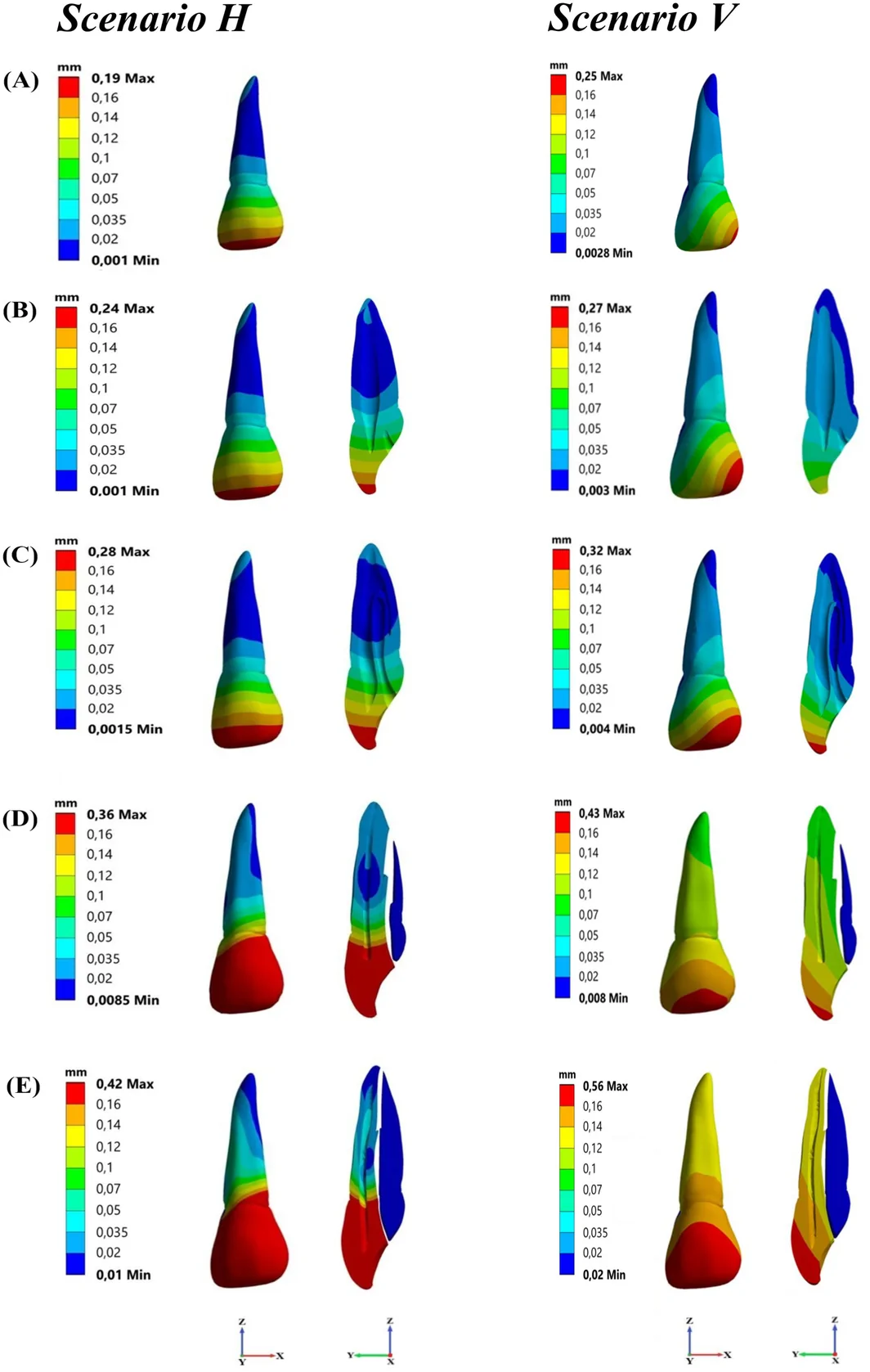
Total deformation distributions in the tooth models under horizontal *(Scenario H)* and vertical *(Scenario V)* impact loading applied to the buccal surface: **(A)** Tooth with normal morphology, **(B)** Type I dens invaginatus, **(C)** Type II dens invaginatus, **(D)** Type IIIa dens invaginatus, **(E)** Type IIIb dens invaginatus.

The time‐dependent microstrain responses obtained under horizontal and vertical impact loading are presented in Figure 8. Comparative analysis demonstrated that the dens invaginatus models exhibited higher microstrain values than the tooth with normal morphology, with vertical impact loading producing greater strain magnitudes overall. Peak microstrain values in the normally structured tooth remained low (approximately 10–20 με), whereas the dens invaginatus models showed substantially higher responses. Under horizontal loading, peak microstrain increased progressively from roughly 100 με in the Type I model to about 140 με in the Type IIIb model. Under vertical loading, peak values further increased, reaching approximately 160 με in the Type IIIb configuration. These findings indicate that increasing severity of dens invaginatus is associated with a marked amplification of the dynamic strain response.

**FIGURE 8:**
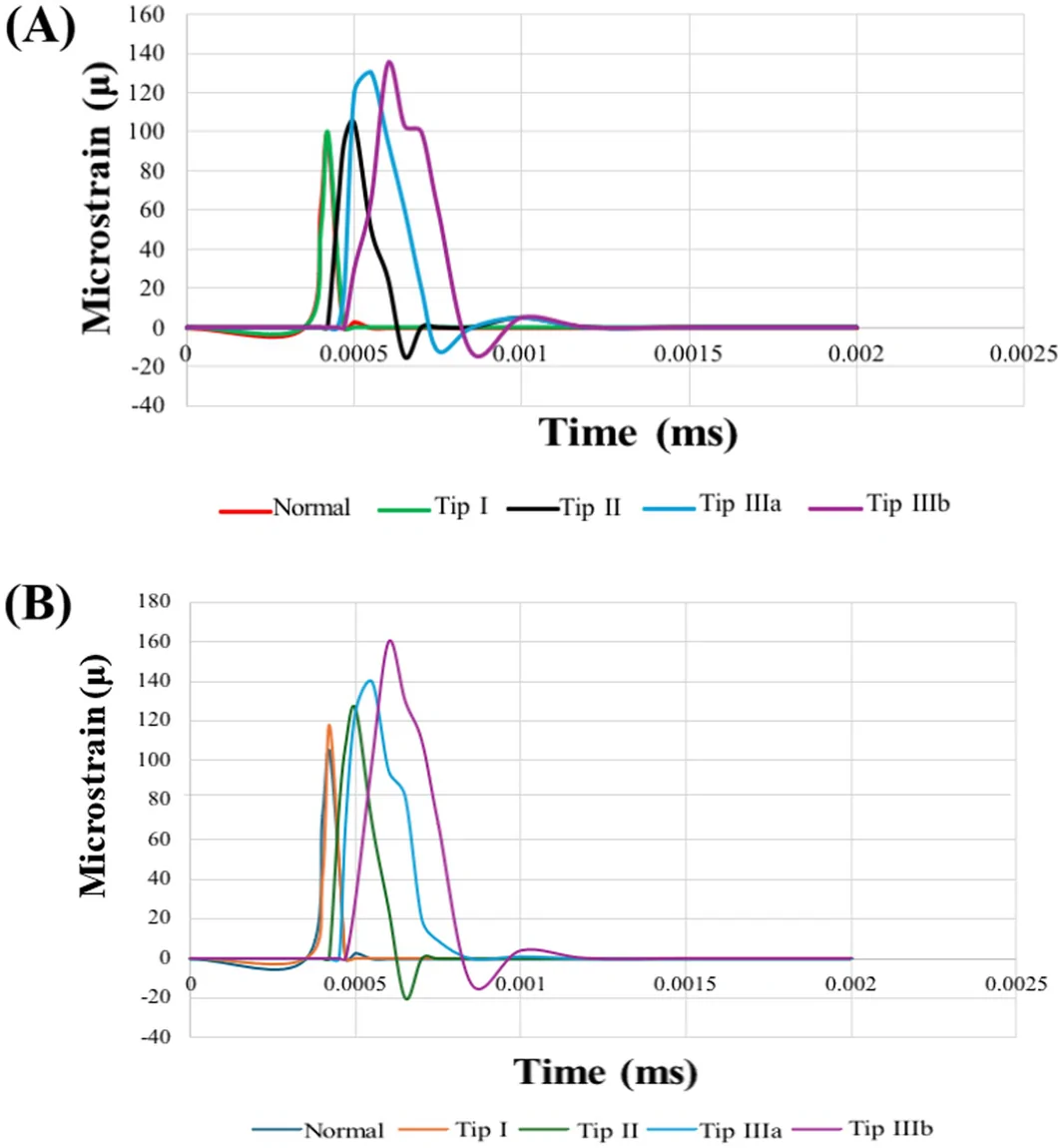
Time‐history plots of microstrain values recorded in the tooth models under horizontal *(Scenario H)* **(A)** and vertical *(Scenario V)* **(B)** impact loading.

## 4 Discussion

The present analysis provides insight into the biomechanical behavior of permanent lateral incisors presenting four different configurations of dens invaginatus under traumatic impact loading, as assessed by FEA. The results demonstrated that stress distribution, deformation magnitude, and strain responses varied substantially according to both loading direction and morphological configuration. While the lowest stress and deformation values were observed in the tooth with normal morphology, the Type IIIb dens invaginatus model exhibited the highest mechanical responses. Accordingly, the null hypothesis that no differences would exist among dens invaginatus configurations and the normally structured tooth was rejected.

Dental trauma, which occurs suddenly and unpredictably, represents a significant clinical challenge that often requires urgent intervention. Its high prevalence during childhood underscores the need for increased clinical awareness [27]. The consequences of traumatic dental injuries depend on multiple variables, including not only environmental factors but also morphological characteristics of the teeth and surrounding tissues [11]. Developmental dental anomalies may therefore increase susceptibility to trauma by altering internal stress distribution and force transmission patterns [28]. Dens invaginatus, characterized by the invagination of enamel and dentin toward the pulp or by inward folding of the palatal pit prior to calcification, results in marked deviations from normal tooth anatomy [6]. The present findings support the view that such structural irregularities may substantially influence biomechanical response to traumatic loading and should therefore be considered during clinical evaluation and treatment planning [28].

Previous experimental investigations of dental trauma have often relied on in vivo animal models, which are costly and raise ethical concerns [29]. Human in vivo trauma studies are not feasible for ethical reasons and long‐term retrospective investigations remain difficult due to follow‐up limitations [25]. Dynamic finite element simulations have therefore emerged as a valuable alternative, as they more realistically reproduce short‐duration traumatic loading conditions than static analyses [30, 31, 32]. Although static models require lower computational resources and may provide comparable results in certain contexts [33, 34, 35], dynamic analysis was preferred in the present study to more accurately simulate the biomechanical effects of trauma.

In *Scenario H*; previous FEA studies by Olsen [36] and Silva et al. [37] reported that the highest stress values tend to occur under horizontally applied traumatic loads. In contrast, the present findings indicate that vertically directed impacts generated greater von Mises stresses, deformation magnitudes, and microstrain values across all models. In particular, maximum stress values increased from approximately 76–78 MPa in the normally structured tooth to nearly 179 MPa in the Type IIIb model under vertical loading, highlighting the dominant mechanical influence of impact direction. These observations are consistent with the results of Huang et al. [31] and Kurt et al. [26], who also reported that vertically oriented traumatic forces produce greater stress accumulation within dental structures. Collectively, these findings reinforce the notion that the direction of traumatic loading is a critical determinant of biomechanical response. Notably, even the Type I dens invaginatus model demonstrated higher stress, deformation, and microstrain values than the normally structured tooth, indicating that biomechanical alterations may arise even in the mildest forms of the anomaly.

Regarding stress distribution patterns, previous studies have demonstrated that horizontally applied forces tend to concentrate stresses in the cervical region of teeth. The present analysis similarly revealed that horizontal impacts produced stress accumulation predominantly in the cervical and middle thirds of the root, whereas vertical loading resulted in more pronounced cervical stress concentration and higher overall stress magnitudes. These distribution patterns correspond well with the observations of Silva et al. [37] and Verissimo et al. [35], who reported elevated stresses in the cervical and middle root regions under horizontal loading conditions. However, the current results further demonstrate that increasing severity of dens invaginatus markedly amplifies these effects, with the Type IIIb configuration exhibiting the highest stress, deformation, and strain responses under both loading directions.

In *Scenario V*; when vertically directed traumatic forces were analyzed in detail, peak stress values were localized at the incisal contact point and in the cervicobuccal region of the alveolar bone along the buccal root surface. This distribution is consistent with the results of Dezzen‐Gomide et al. [33] and Silva et al. [37], who demonstrated pronounced stress accumulation in the buccal alveolar region under vertical loading. In an avulsion simulation, Miura and Maeda [30] showed that buccally directed forces produced peak stresses in the labial alveolus until periodontal ligament rupture occurred, after which stress redistribution toward the palatal side was observed. Similarly, Huang et al. [31] reported that vertically applied traumatic forces may result in fracture formation in the buccal wall of the alveolar bone and highlighted the destructive potential of vertically directed trauma on alveolar structural integrity. Collectively, these observations support the present results and indicate that traumatic forces acting along the vertical axis generate substantial biomechanical stresses, particularly in the labial and cervicobuccal regions.

Vertically applied forces have also been associated with enamel damage. Jayasudha et al. [38] reported that incisal impacts may produce cracks confined to the cervical enamel, while Huang et al. [31] suggested that stress levels exceeding approximately 50 MPa represent a critical threshold for enamel fracture, especially on the buccal surface. In the present study, von Mises stress values under vertical loading ranged from approximately 78 MPa to nearly 180 MPa, clearly exceeding the fracture thresholds reported in the literature. These findings suggest that vertically directed traumatic forces may significantly compromise enamel integrity. Importantly, anatomical variations such as dens invaginatus further amplified this biomechanical vulnerability [28]. Even Type I dens invaginatus, the most frequently observed form [5], exhibited higher stress and deformation values than a tooth with normal morphology, indicating that biomechanical alterations may arise even at the early stages of the anomaly.

Deformation analysis further demonstrated that, under both loading conditions, maximum deformation occurred at the impact site and gradually decreased with increasing distance from the contact region. This pattern is consistent with the observations of Kurt et al. [26] Similarly, Vilela et al. [25], who similarly reported peak displacement at the point of traumatic contact with diminishing effects toward the root. Together, these findings suggest that traumatic loading induces localized structural responses that propagate through the tooth–bone complex, with deformation magnitude strongly influenced by both loading direction and morphological configuration. Overall, the results highlight that dens invaginatus acts as a biomechanical risk modifier that may increase structural vulnerability even in its mildest forms.

Beyond the biomechanical findings, the present results have important clinical implications. The findings suggest that dens invaginatus may increase susceptibility to traumatic dental injuries by altering internal stress distribution and amplifying deformation responses. Notably, elevated stress levels observed across all configurations, including the mildest form (Type I), indicate that even subtle morphological alterations may predispose teeth to structural damage under traumatic loading. These observations may directly inform clinical decision‐making. Teeth with dens invaginatus may warrant closer monitoring following traumatic events, even in the absence of immediate clinical signs. In addition, increased stress concentrations in the cervical and enamel regions suggest a higher risk of crown fractures and enamel cracks, whereas elevated strain values may be associated with a greater likelihood of periodontal or root‐related injuries. Accordingly, dens invaginatus should be considered not only as an anatomical anomaly but also as a biomechanical factor that may influence trauma severity, prognosis, and treatment planning. Incorporating this perspective into clinical evaluation may support more individualized and preventive approaches in dental trauma management.

This study has several limitations related to the finite element analysis methodology. Although enamel, dentin, and bone exhibit anisotropic and heterogeneous mechanical behavior under physiological conditions, all materials were modeled as isotropic and homogeneous to ensure computational feasibility and analytical consistency. Consequently, certain generalizations and assumptions were required, and calculations were performed using average mechanical property values. Previous studies have reported that such assumptions do not substantially influence overall stress distribution patterns and that the resulting simulations remain largely consistent with clinical observations [39, 40]. Nevertheless, the inability of finite element analysis to fully reproduce the complex mechanical behavior of living tissues represents an inherent limitation that should be considered when interpreting the findings.

## 5 Conclusion

Finite element impact analysis demonstrated that both tooth morphology and impact direction play critical roles in determining biomechanical response under traumatic loading. Vertically directed forces generated greater stress accumulation and deformation than horizontal loading, emphasizing the dominant influence of impact direction in trauma biomechanics. The presence of dens invaginatus markedly altered stress distribution and amplified mechanical responses, indicating that this anomaly may be considered not only as a morphological variation but also as a potential biomechanical risk factor influencing trauma susceptibility and treatment planning. Finite element impact modeling therefore offers a valuable quantitative tool for understanding the biomechanical consequences of anatomical variations and may support more informed, patient‐specific clinical decision‐making in dental trauma management.

## Author Contributions

The study concept and manuscript drafting were performed by Sema Aydınoğlu, Emine Şuranur Ayaz, and Dilara Nil Günaçar. The Materials and Methods design, data curation, formal analysis, and technical modeling were carried out by the co‐authors. All authors contributed to the interpretation of the results, critically revised the manuscript for intellectual content, and approved the final version.

## Funding

The authors have nothing to report.

## Conflicts of Interest

The authors declare no conflicts of interest.

## Data Availability

The data that support the findings of this study are available from the corresponding author upon reasonable request.
